# Supplementary figures and images for: Metagenomic insight into taxonomic composition, environmental filtering and functional redundancy for shaping worldwide modern non-lithifying microbial mats
Source: PeerJ. 2024 May 30;12:e17412. doi: 10.7717/peerj.17412 (PMC11144394; doi:10.7717/peerj.17412)

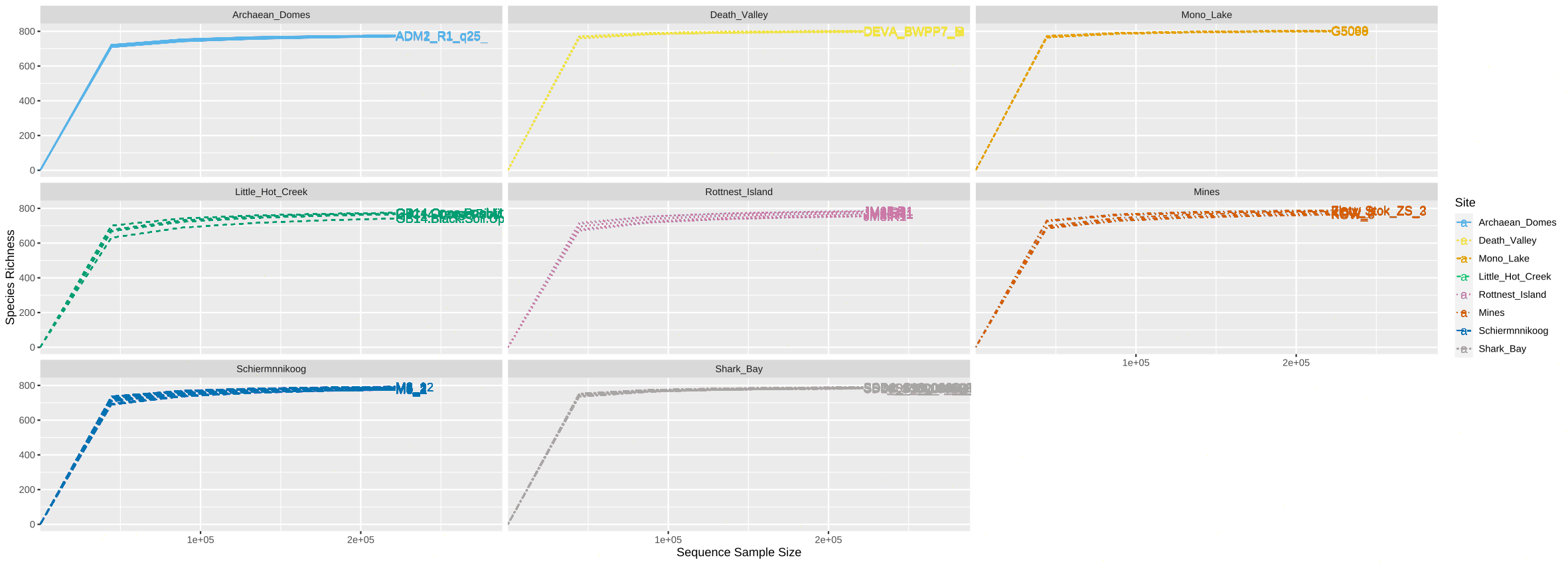

Supplement: Supplemental Information 1 — Each site is depicted in its own chart. Sequence sample size refers to the amount of reads from shotgun metagenomic sequencing. [file peerj-12-17412-s001.png]

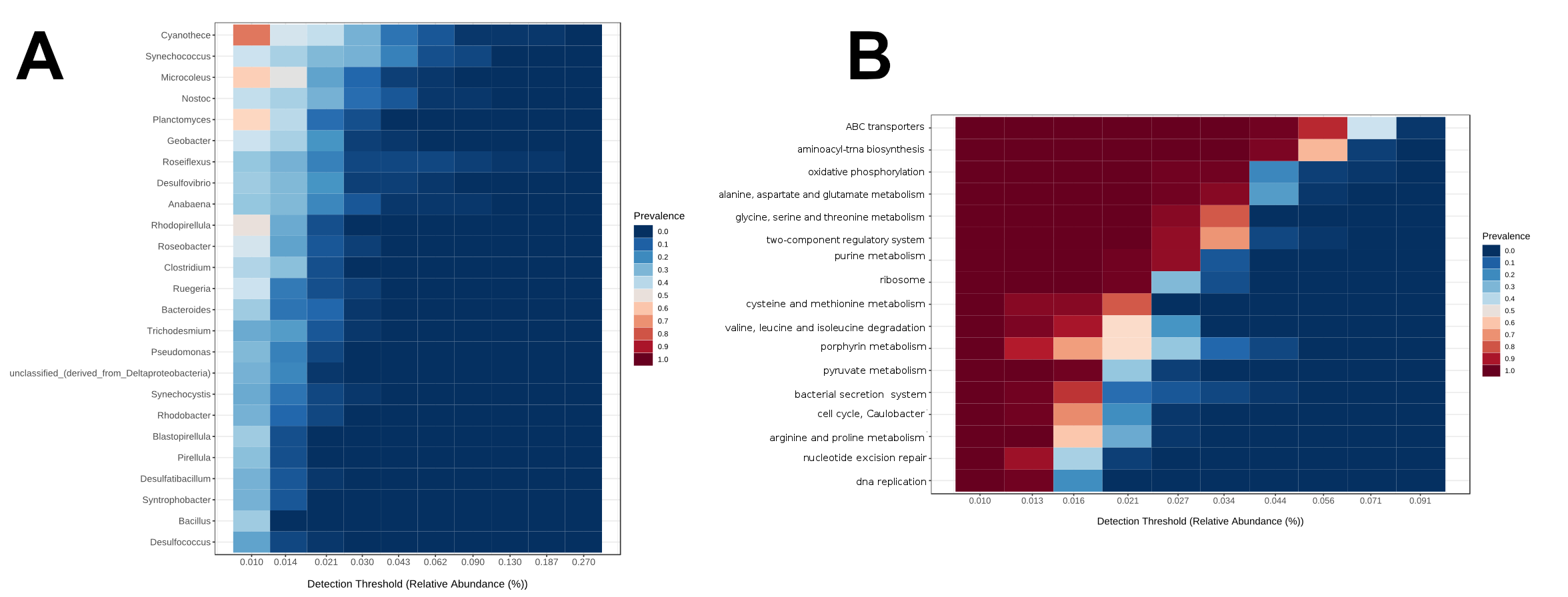

Supplement: Supplemental Information 2 — Prevalence refers to the portion of the total metagenomes on which a particular A) taxa or B) function was found. Detection threshold refers to the relative abundance that particular taxa or function was found. [file peerj-12-17412-s002.png]

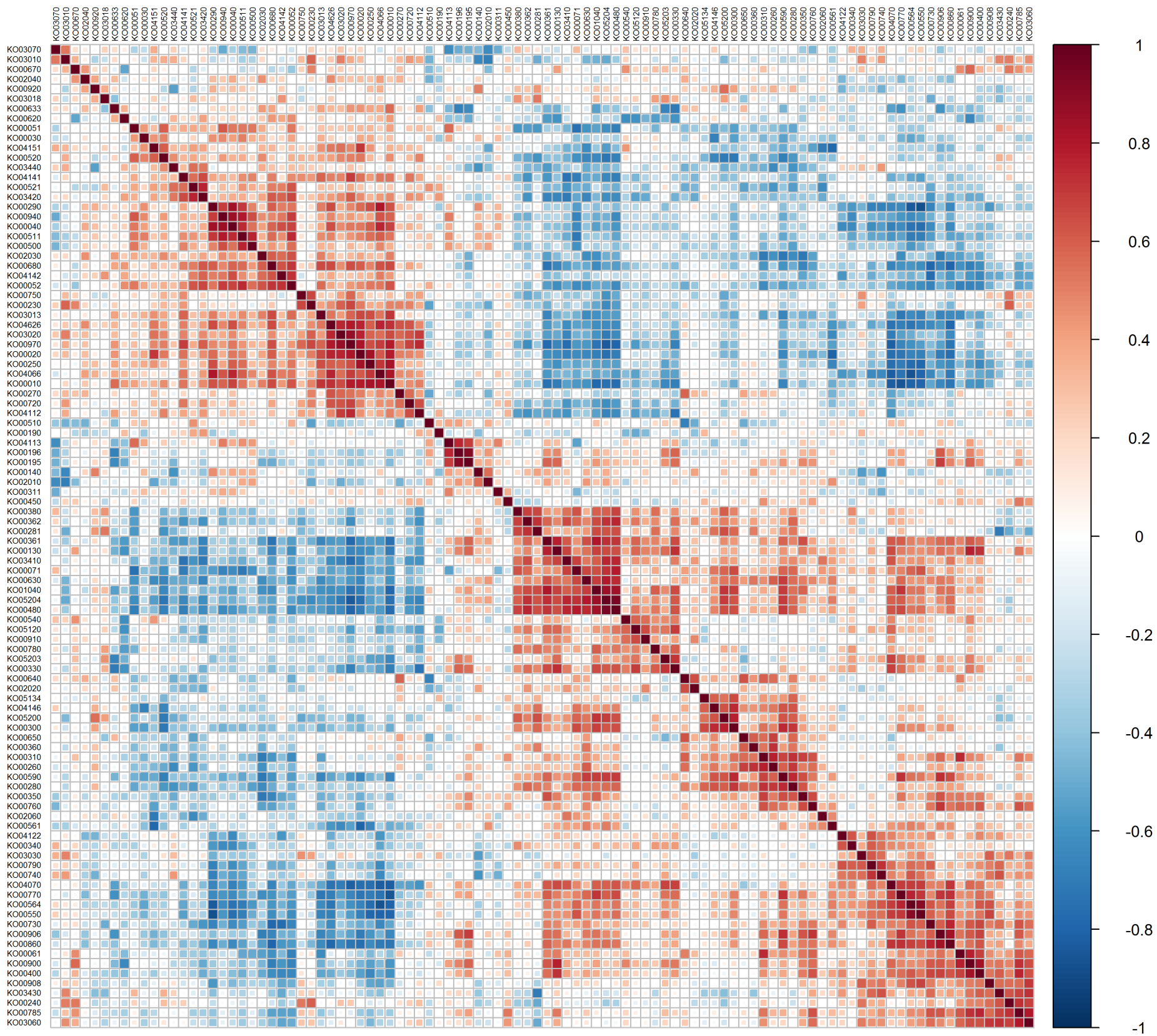

Supplement: Supplemental Information 3 — Spearman’s correlation test was performed on the relative abundance of the top 100 most abundant functions (KEGG Orthology). [file peerj-12-17412-s003.pdf]
