# Supplementary material for: Metagenomic insight into taxonomic composition, environmental filtering and functional redundancy for shaping worldwide modern non-lithifying microbial mats
Source: PeerJ. 2024 May 30;12:e17412. doi: 10.7717/peerj.17412 (PMC11144394; doi:10.7717/peerj.17412)
Supplement: Supplemental Information 4 [file peerj-12-17412-s004.doc]

|  | **Temperature (°C)** | **pH** | **Publication** | **Notes** |
| --- | --- | --- | --- | --- |
| Kowary | 10,5 | 5,83 | Drewniak et al., 2016 (based on highest and lowest) | measurements taken from water samples |
|  | 10,5 | 6 |  |  |
| Zloty_Stok | 10,2 | 7,59 |  |  |
|  | 10,2 | 8,01 |  |  |
| Archaean_Domes | 32,45 | 9,94 | This study | measurements taken from water samples |
| Little_Hot_Creek | 81,2 | 6,7 | Kraus et al., 2018 | measurements taken from water samples |
|  | 79,6 | 6,8 |  |  |
|  | 76,7 | 7,07 |  |  |
|  | 70,7 | 7,45 |  |  |
| Shark_Bay | 22 | 8 | Ruvindy et al., 2015 (based on highest and lowest) | measurements taken from water samples |
|  | 25 | 8 |  |  |
| Schiermonnikoog | 17 | 8.7±0.1 | Temperature from Fan et al., 2015 (yearly cycle of July 2010 to April 2011); pH by Dini-Andreote et al., 2016 (July 2012) | measurements taken from sediment |
|  | 10 | 8.3±0.1 |  |  |
|  | 9 | 7.8±0.1 |  |  |
|  | 0 | 7.4±0.1 |  |  |
|  | 8 | 7.4±0.1 |  |  |
| Mono Lake | 7 | 9,3 | Temperature from Stamp et al., 2018; pH from mgp19115 | measurements taken from water samples |
|  | 15 | 9,07 |  |  |
| Death Valley | 17,6 | 8,04 | mgp19115 | measurements taken from water samples |
| Rottnest Island | NA | 7,8 | pH from Mendes Monteiro et al., 2020 | measurements taken from water samples |
|  | NA | 7,79 |  |  |
|  | NA | 8,06 |  |  |
|  | NA | 7,82 |  |  |
