# Supplementary material for: Metagenomic insight into taxonomic composition, environmental filtering and functional redundancy for shaping worldwide modern non-lithifying microbial mats
Source: PeerJ. 2024 May 30;12:e17412. doi: 10.7717/peerj.17412 (PMC11144394; doi:10.7717/peerj.17412)
Supplement: Supplemental Information 5 [file peerj-12-17412-s005.doc]

| 00010 Glycolysis / Gluconeogenesis [PATH:ko00010] |
| --- |
| 00020 Citrate cycle (TCA cycle) [PATH:ko00020] |
| 00030 Pentose phosphate pathway [PATH:ko00030] |
| 00040 Pentose and glucuronate interconversions [PATH:ko00040] |
| 00051 Fructose and mannose metabolism [PATH:ko00051] |
| 00052 Galactose metabolism [PATH:ko00052] |
| 00053 Ascorbate and aldarate metabolism [PATH:ko00053] |
| 00061 Fatty acid biosynthesis [PATH:ko00061] |
| 00062 Fatty acid elongation [PATH:ko00062] |
| 00071 Fatty acid metabolism [PATH:ko00071] |
| 00100 Steroid biosynthesis [PATH:ko00100] |
| 00120 Primary bile acid biosynthesis [PATH:ko00120] |
| 00130 Ubiquinone and other terpenoid-quinone biosynthesis [PATH:ko00130] |
| 00140 Steroid hormone biosynthesis [PATH:ko00140] |
| 00190 Oxidative phosphorylation [PATH:ko00190] |
| 00195 Photosynthesis [PATH:ko00195] |
| 00196 Photosynthesis - antenna proteins [PATH:ko00196] |
| 00230 Purine metabolism [PATH:ko00230] |
| 00232 Caffeine metabolism [PATH:ko00232] |
| 00240 Pyrimidine metabolism [PATH:ko00240] |
| 00250 Alanine, aspartate and glutamate metabolism [PATH:ko00250] |
| 00253 Tetracycline biosynthesis [PATH:ko00253] |
| 00260 Glycine, serine and threonine metabolism [PATH:ko00260] |
| 00270 Cysteine and methionine metabolism [PATH:ko00270] |
| 00280 Valine, leucine and isoleucine degradation [PATH:ko00280] |
| 00281 Geraniol degradation [PATH:ko00281] |
| 00290 Valine, leucine and isoleucine biosynthesis [PATH:ko00290] |
| 00300 Lysine biosynthesis [PATH:ko00300] |
| 00310 Lysine degradation [PATH:ko00310] |
| 00311 Penicillin and cephalosporin biosynthesis [PATH:ko00311] |
| 00330 Arginine and proline metabolism [PATH:ko00330] |
| 00340 Histidine metabolism [PATH:ko00340] |
| 00350 Tyrosine metabolism [PATH:ko00350] |
| 00351 1,1,1-Trichloro-2,2-bis(4-chlorophenyl)ethane (DDT) degradation [PATH:ko00351] |
| 00360 Phenylalanine metabolism [PATH:ko00360] |
| 00361 Chlorocyclohexane and chlorobenzene degradation [PATH:ko00361] |
| 00362 Benzoate degradation [PATH:ko00362] |
| 00363 Bisphenol degradation [PATH:ko00363] |
| 00364 Fluorobenzoate degradation [PATH:ko00364] |
| 00380 Tryptophan metabolism [PATH:ko00380] |
| 00400 Phenylalanine, tyrosine and tryptophan biosynthesis [PATH:ko00400] |
| 00410 beta-Alanine metabolism [PATH:ko00410] |
| 00430 Taurine and hypotaurine metabolism [PATH:ko00430] |
| 00440 Phosphonate and phosphinate metabolism [PATH:ko00440] |
| 00450 Selenocompound metabolism [PATH:ko00450] |
| 00460 Cyanoamino acid metabolism [PATH:ko00460] |
| 00480 Glutathione metabolism [PATH:ko00480] |
| 00500 Starch and sucrose metabolism [PATH:ko00500] |
| 00510 N-Glycan biosynthesis [PATH:ko00510] |
| 00511 Other glycan degradation [PATH:ko00511] |
| 00512 Mucin type O-glycan biosynthesis [PATH:ko00512] |
| 00513 Various types of N-glycan biosynthesis [PATH:ko00513] |
| 00514 Other types of O-glycan biosynthesis [PATH:ko00514] |
| 00520 Amino sugar and nucleotide sugar metabolism [PATH:ko00520] |
| 00521 Streptomycin biosynthesis [PATH:ko00521] |
| 00522 Biosynthesis of 12-, 14- and 16-membered macrolides [PATH:ko00522] |
| 00531 Glycosaminoglycan degradation [PATH:ko00531] |
| 00532 Glycosaminoglycan biosynthesis - chondroitin sulfate / dermatan sulfate [PATH:ko00532] |
| 00534 Glycosaminoglycan biosynthesis - heparan sulfate / heparin [PATH:ko00534] |
| 00540 Lipopolysaccharide biosynthesis [PATH:ko00540] |
| 00550 Peptidoglycan biosynthesis [PATH:ko00550] |
| 00561 Glycerolipid metabolism [PATH:ko00561] |
| 00562 Inositol phosphate metabolism [PATH:ko00562] |
| 00563 Glycosylphosphatidylinositol(GPI)-anchor biosynthesis [PATH:ko00563] |
| 00564 Glycerophospholipid metabolism [PATH:ko00564] |
| 00565 Ether lipid metabolism [PATH:ko00565] |
| 00590 Arachidonic acid metabolism [PATH:ko00590] |
| 00591 Linoleic acid metabolism [PATH:ko00591] |
| 00592 alpha-Linolenic acid metabolism [PATH:ko00592] |
| 00600 Sphingolipid metabolism [PATH:ko00600] |
| 00601 Glycosphingolipid biosynthesis - lacto and neolacto series [PATH:ko00601] |
| 00620 Pyruvate metabolism [PATH:ko00620] |
| 00621 Dioxin degradation [PATH:ko00621] |
| 00622 Xylene degradation [PATH:ko00622] |
| 00623 Toluene degradation [PATH:ko00623] |
| 00624 Polycyclic aromatic hydrocarbon degradation [PATH:ko00624] |
| 00625 Chloroalkane and chloroalkene degradation [PATH:ko00625] |
| 00627 Aminobenzoate degradation [PATH:ko00627] |
| 00630 Glyoxylate and dicarboxylate metabolism [PATH:ko00630] |
| 00633 Nitrotoluene degradation [PATH:ko00633] |
| 00640 Propanoate metabolism [PATH:ko00640] |
| 00642 Ethylbenzene degradation [PATH:ko00642] |
| 00643 Styrene degradation [PATH:ko00643] |
| 00650 Butanoate metabolism [PATH:ko00650] |
| 00660 C5-Branched dibasic acid metabolism [PATH:ko00660] |
| 00670 One carbon pool by folate [PATH:ko00670] |
| 00680 Methane metabolism [PATH:ko00680] |
| 00710 Carbon fixation in photosynthetic organisms [PATH:ko00710] |
| 00720 Carbon fixation pathways in prokaryotes [PATH:ko00720] |
| 00730 Thiamine metabolism [PATH:ko00730] |
| 00740 Riboflavin metabolism [PATH:ko00740] |
| 00750 Vitamin B6 metabolism [PATH:ko00750] |
| 00760 Nicotinate and nicotinamide metabolism [PATH:ko00760] |
| 00770 Pantothenate and CoA biosynthesis [PATH:ko00770] |
| 00780 Biotin metabolism [PATH:ko00780] |
| 00785 Lipoic acid metabolism [PATH:ko00785] |
| 00790 Folate biosynthesis [PATH:ko00790] |
| 00791 Atrazine degradation [PATH:ko00791] |
| 00830 Retinol metabolism [PATH:ko00830] |
| 00860 Porphyrin and chlorophyll metabolism [PATH:ko00860] |
| 00900 Terpenoid backbone biosynthesis [PATH:ko00900] |
| 00901 Indole alkaloid biosynthesis [PATH:ko00901] |
| 00903 Limonene and pinene degradation [PATH:ko00903] |
| 00904 Diterpenoid biosynthesis [PATH:ko00904] |
| 00906 Carotenoid biosynthesis [PATH:ko00906] |
| 00908 Zeatin biosynthesis [PATH:ko00908] |
| 00909 Sesquiterpenoid and triterpenoid biosynthesis [PATH:ko00909] |
| 00910 Nitrogen metabolism [PATH:ko00910] |
| 00920 Sulfur metabolism [PATH:ko00920] |
| 00930 Caprolactam degradation [PATH:ko00930] |
| 00940 Phenylpropanoid biosynthesis [PATH:ko00940] |
| 00941 Flavonoid biosynthesis [PATH:ko00941] |
| 00943 Isoflavonoid biosynthesis [PATH:ko00943] |
| 00945 Stilbenoid, diarylheptanoid and gingerol biosynthesis [PATH:ko00945] |
| 00960 Tropane, piperidine and pyridine alkaloid biosynthesis [PATH:ko00960] |
| 00970 Aminoacyl-tRNA biosynthesis [PATH:ko00970] |
| 00983 Drug metabolism - other enzymes [PATH:ko00983] |
| 00984 Steroid degradation [PATH:ko00984] |
| 01040 Biosynthesis of unsaturated fatty acids [PATH:ko01040] |
| 01053 Biosynthesis of siderophore group nonribosomal peptides [PATH:ko01053] |
| 01056 Biosynthesis of type II polyketide backbone [PATH:ko01056] |
| 01057 Biosynthesis of type II polyketide products [PATH:ko01057] |
| 02010 ABC transporters [PATH:ko02010] |
| 02020 Two-component system [PATH:ko02020] |
| 02030 Bacterial chemotaxis [PATH:ko02030] |
| 02040 Flagellar assembly [PATH:ko02040] |
| 02060 Phosphotransferase system (PTS) [PATH:ko02060] |
| 03008 Ribosome biogenesis in eukaryotes [PATH:ko03008] |
| 03010 Ribosome [PATH:ko03010] |
| 03013 RNA transport [PATH:ko03013] |
| 03015 mRNA surveillance pathway [PATH:ko03015] |
| 03018 RNA degradation [PATH:ko03018] |
| 03020 RNA polymerase [PATH:ko03020] |
| 03022 Basal transcription factors [PATH:ko03022] |
| 03030 DNA replication [PATH:ko03030] |
| 03040 Spliceosome [PATH:ko03040] |
| 03050 Proteasome [PATH:ko03050] |
| 03060 Protein export [PATH:ko03060] |
| 03070 Bacterial secretion system [PATH:ko03070] |
| 03320 PPAR signaling pathway [PATH:ko03320] |
| 03410 Base excision repair [PATH:ko03410] |
| 03420 Nucleotide excision repair [PATH:ko03420] |
| 03430 Mismatch repair [PATH:ko03430] |
| 03440 Homologous recombination [PATH:ko03440] |
| 03450 Non-homologous end-joining [PATH:ko03450] |
| 04010 MAPK signaling pathway [PATH:ko04010] |
| 04011 MAPK signaling pathway - yeast [PATH:ko04011] |
| 04012 ErbB signaling pathway [PATH:ko04012] |
| 04013 MAPK signaling pathway - fly [PATH:ko04013] |
| 04020 Calcium signaling pathway [PATH:ko04020] |
| 04060 Cytokine-cytokine receptor interaction [PATH:ko04060] |
| 04064 NF-kappa B signaling pathway [PATH:ko04064] |
| 04066 HIF-1 signaling pathway [PATH:ko04066] |
| 04070 Phosphatidylinositol signaling system [PATH:ko04070] |
| 04075 Plant hormone signal transduction [PATH:ko04075] |
| 04080 Neuroactive ligand-receptor interaction [PATH:ko04080] |
| 04110 Cell cycle [PATH:ko04110] |
| 04111 Cell cycle - yeast [PATH:ko04111] |
| 04112 Cell cycle - Caulobacter [PATH:ko04112] |
| 04113 Meiosis - yeast [PATH:ko04113] |
| 04114 Oocyte meiosis [PATH:ko04114] |
| 04115 p53 signaling pathway [PATH:ko04115] |
| 04120 Ubiquitin mediated proteolysis [PATH:ko04120] |
| 04122 Sulfur relay system [PATH:ko04122] |
| 04130 SNARE interactions in vesicular transport [PATH:ko04130] |
| 04140 Regulation of autophagy [PATH:ko04140] |
| 04141 Protein processing in endoplasmic reticulum [PATH:ko04141] |
| 04142 Lysosome [PATH:ko04142] |
| 04144 Endocytosis [PATH:ko04144] |
| 04145 Phagosome [PATH:ko04145] |
| 04146 Peroxisome [PATH:ko04146] |
| 04150 mTOR signaling pathway [PATH:ko04150] |
| 04151 PI3K-Akt signaling pathway [PATH:ko04151] |
| 04210 Apoptosis [PATH:ko04210] |
| 04260 Cardiac muscle contraction [PATH:ko04260] |
| 04310 Wnt signaling pathway [PATH:ko04310] |
| 04320 Dorso-ventral axis formation [PATH:ko04320] |
| 04330 Notch signaling pathway [PATH:ko04330] |
| 04340 Hedgehog signaling pathway [PATH:ko04340] |
| 04350 TGF-beta signaling pathway [PATH:ko04350] |
| 04360 Axon guidance [PATH:ko04360] |
| 04510 Focal adhesion [PATH:ko04510] |
| 04512 ECM-receptor interaction [PATH:ko04512] |
| 04514 Cell adhesion molecules (CAMs) [PATH:ko04514] |
| 04520 Adherens junction [PATH:ko04520] |
| 04530 Tight junction [PATH:ko04530] |
| 04540 Gap junction [PATH:ko04540] |
| 04610 Complement and coagulation cascades [PATH:ko04610] |
| 04614 Renin-angiotensin system [PATH:ko04614] |
| 04622 RIG-I-like receptor signaling pathway [PATH:ko04622] |
| 04626 Plant-pathogen interaction [PATH:ko04626] |
| 04630 Jak-STAT signaling pathway [PATH:ko04630] |
| 04670 Leukocyte transendothelial migration [PATH:ko04670] |
| 04710 Circadian rhythm [PATH:ko04710] |
| 04712 Circadian rhythm - plant [PATH:ko04712] |
| 04740 Olfactory transduction [PATH:ko04740] |
| 04744 Phototransduction [PATH:ko04744] |
| 04745 Phototransduction - fly [PATH:ko04745] |
| 04810 Regulation of actin cytoskeleton [PATH:ko04810] |
| 04910 Insulin signaling pathway [PATH:ko04910] |
| 04940 Type I diabetes mellitus [PATH:ko04940] |
| 04950 Maturity onset diabetes of the young [PATH:ko04950] |
| 04962 Vasopressin-regulated water reabsorption [PATH:ko04962] |
| 04966 Collecting duct acid secretion [PATH:ko04966] |
| 04970 Salivary secretion [PATH:ko04970] |
| 04972 Pancreatic secretion [PATH:ko04972] |
| 04973 Carbohydrate digestion and absorption [PATH:ko04973] |
| 04974 Protein digestion and absorption [PATH:ko04974] |
| 04975 Fat digestion and absorption [PATH:ko04975] |
| 04976 Bile secretion [PATH:ko04976] |
| 05010 Alzheimer's disease [PATH:ko05010] |
| 05012 Parkinson's disease [PATH:ko05012] |
| 05014 Amyotrophic lateral sclerosis (ALS) [PATH:ko05014] |
| 05016 Huntington's disease [PATH:ko05016] |
| 05020 Prion diseases [PATH:ko05020] |
| 05030 Cocaine addiction [PATH:ko05030] |
| 05032 Morphine addiction [PATH:ko05032] |
| 05034 Alcoholism [PATH:ko05034] |
| 05100 Bacterial invasion of epithelial cells [PATH:ko05100] |
| 05110 Vibrio cholerae infection [PATH:ko05110] |
| 05111 Vibrio cholerae pathogenic cycle [PATH:ko05111] |
| 05120 Epithelial cell signaling in Helicobacter pylori infection [PATH:ko05120] |
| 05132 Salmonella infection [PATH:ko05132] |
| 05133 Pertussis [PATH:ko05133] |
| 05134 Legionellosis [PATH:ko05134] |
| 05140 Leishmaniasis [PATH:ko05140] |
| 05142 Chagas disease (American trypanosomiasis) [PATH:ko05142] |
| 05143 African trypanosomiasis [PATH:ko05143] |
| 05144 Malaria [PATH:ko05144] |
| 05145 Toxoplasmosis [PATH:ko05145] |
| 05146 Amoebiasis [PATH:ko05146] |
| 05150 Staphylococcus aureus infection [PATH:ko05150] |
| 05152 Tuberculosis [PATH:ko05152] |
| 05168 Herpes simplex infection [PATH:ko05168] |
| 05200 Pathways in cancer [PATH:ko05200] |
| 05202 Transcriptional misregulation in cancers [PATH:ko05202] |
| 05203 Viral carcinogenesis [PATH:ko05203] |
| 05204 Chemical carcinogenesis [PATH:ko05204] |
| 05215 Prostate cancer [PATH:ko05215] |
| 05219 Bladder cancer [PATH:ko05219] |
| 05320 Autoimmune thyroid disease [PATH:ko05320] |
| 05322 Systemic lupus erythematosus [PATH:ko05322] |
| 05340 Primary immunodeficiency [PATH:ko05340] |
| 05410 Hypertrophic cardiomyopathy (HCM) [PATH:ko05410] |
| 00312 beta-Lactam resistance [PATH:ko00312] |
| 00523 Polyketide sugar unit biosynthesis [PATH:ko00523] |
| 00532 Glycosaminoglycan biosynthesis - chondroitin sulfate [PATH:ko00532] |
| 00533 Glycosaminoglycan biosynthesis - keratan sulfate [PATH:ko00533] |
| 00604 Glycosphingolipid biosynthesis - ganglio series [PATH:ko00604] |
| 00905 Brassinosteroid biosynthesis [PATH:ko00905] |
| 00944 Flavone and flavonol biosynthesis [PATH:ko00944] |
| 04062 Chemokine signaling pathway [PATH:ko04062] |
| 04270 Vascular smooth muscle contraction [PATH:ko04270] |
| 04380 Osteoclast differentiation [PATH:ko04380] |
| 04390 Hippo signaling pathway [PATH:ko04390] |
| 04612 Antigen processing and presentation [PATH:ko04612] |
| 04620 Toll-like receptor signaling pathway [PATH:ko04620] |
| 04621 NOD-like receptor signaling pathway [PATH:ko04621] |
| 04666 Fc gamma R-mediated phagocytosis [PATH:ko04666] |
| 04711 Circadian rhythm - fly [PATH:ko04711] |
| 04722 Neurotrophin signaling pathway [PATH:ko04722] |
| 04723 Retrograde endocannabinoid signaling [PATH:ko04723] |
| 04742 Taste transduction [PATH:ko04742] |
| 04920 Adipocytokine signaling pathway [PATH:ko04920] |
| 04930 Type II diabetes mellitus [PATH:ko04930] |
| 04964 Proximal tubule bicarbonate reclamation [PATH:ko04964] |
| 04971 Gastric acid secretion [PATH:ko04971] |
| 05130 Pathogenic Escherichia coli infection [PATH:ko05130] |
| 05131 Shigellosis [PATH:ko05131] |
| 05160 Hepatitis C [PATH:ko05160] |
| 05161 Hepatitis B [PATH:ko05161] |
| 05164 Influenza A [PATH:ko05164] |
| 05166 HTLV-I infection [PATH:ko05166] |
| 05169 Epstein-Barr virus infection [PATH:ko05169] |
| 05222 Small cell lung cancer [PATH:ko05222] |
| 05310 Asthma [PATH:ko05310] |
| 05323 Rheumatoid arthritis [PATH:ko05323] |
| 00331 Clavulanic acid biosynthesis [PATH:ko00331] |
| 00981 Insect hormone biosynthesis [PATH:ko00981] |
| 04912 GnRH signaling pathway [PATH:ko04912] |
| 05211 Renal cell carcinoma [PATH:ko05211] |
| 04662 B cell receptor signaling pathway [PATH:ko04662] |
| 04730 Long-term depression [PATH:ko04730] |
| 05162 Measles [PATH:ko05162] |
| 05221 Acute myeloid leukemia [PATH:ko05221] |
| 04623 Cytosolic DNA-sensing pathway [PATH:ko04623] |
| 00603 Glycosphingolipid biosynthesis - globo series [PATH:ko00603] |
| 00980 Metabolism of xenobiotics by cytochrome P450 [PATH:ko00980] |
| 04370 VEGF signaling pathway [PATH:ko04370] |
| 04640 Hematopoietic cell lineage [PATH:ko04640] |
| 04650 Natural killer cell mediated cytotoxicity [PATH:ko04650] |
| 04660 T cell receptor signaling pathway [PATH:ko04660] |
| 04672 Intestinal immune network for IgA production [PATH:ko04672] |
| 04960 Aldosterone-regulated sodium reabsorption [PATH:ko04960] |
| 05220 Chronic myeloid leukemia [PATH:ko05220] |
| 05412 Arrhythmogenic right ventricular cardiomyopathy (ARVC) [PATH:ko05412] |
