# Supplementary material for: Metagenomic insight into taxonomic composition, environmental filtering and functional redundancy for shaping worldwide modern non-lithifying microbial mats
Source: PeerJ. 2024 May 30;12:e17412. doi: 10.7717/peerj.17412 (PMC11144394; doi:10.7717/peerj.17412)
Supplement: Supplemental Information 6 — Tax: Bray-Curtis distance matrix of taxonomic relative abundances; func: Bray-Curtis distance matrix of functional relative abundances; geo: harvesine distance matrix of geographic coordinates; T+pH: euclidean distance matrix of normalized values of temperature and pH. [file peerj-12-17412-s006.doc]

|  | Mantel's r | Signif |
| --- | --- | --- |
| tax_vs_T+pH | 0.2883 | 145 |
| tax_vs_geo | -0.1189 | 0.7587 |
| func_vs_T+pH | -0.2051 | 0.7887 |
| func_vs_geo | 427 | 0.0111 |
